# Report of an Iranian child with chronic abdominal pain and constipation diagnosed as glycogen storage disease type IX: a case report

**DOI:** 10.1186/s13256-023-04295-0

**Published:** 2024-01-12

**Authors:** Daniel Zamanfar, Seyed MohammadBagher Hashemi-Soteh, Mobin Ghazaiean, Elham Keyhanian

**Affiliations:** 1 Department of Pediatric Endocrinology, Diabetes Reaserch Center of Mazandaran, Mazandaran University of Medical Sciences, Sari, Iran; 2 Immunogenetic Research Center, Molecular and Cell Biology Research Center, Faculty of Medicine, Mazandaran University of Medical Sciences, Sari, Iran; 3 Gut and Liver Research Center, Non-Communicable Disease Institute, Mazandaran University of Medical Sciences, Sari, Iran; 4 Rajaie Cardiovascular Medical and Research Center, Iran University of Medical Science, Tehran, Iran

**Keywords:** Glycogen storage disease type IX, *PHKB*, Liver, Hepatomegaly, Poor growth

## Abstract

**Background:**

Glycogen storage disease type IX is a rare disorder that can cause a wide variety of symptoms depending on the specific deficiency of the phosphorylase kinase enzyme and the organs it affects.

**Case presentation:**

A 4-and-a-half-year-old Caucasian girl was referred to our clinic with a liver biopsy report indicating a diagnosis of glycogen storage disease. Prior to being referred to our clinic, the patient had been under the care of pediatric gastroenterologists. The patient’s initial symptoms included chronic abdominal pain, constipation, and elevated liver transaminase. With the help of the pediatric gastroenterologists, cholestasis, Wilson disease, and autoimmune hepatitis were ruled out. Given that glycogen storage diseases type I and type III are the most common, we initially managed the patient with frequent feedings and a diet that included complex carbohydrates such as a corn starch supplement and a lactose restriction. Following an unfavorable growth velocity and hepatomegaly during the follow-up period, genetic analysis was conducted, which revealed a novel mutation of the phosphorylase kinase regulatory subunit beta gene— a c.C412T (P.Q138x) mutation. As the diagnosis of glycogen storage disease type IX was confirmed, the treatment regimen was altered to a high protein diet (more than 2 g/kg/day) and a low fat diet.

**Conclusion:**

Given the mild and varied clinical manifestations of glycogen storage disease type IX, it is possible for the diagnosis to be overlooked. It is important to consider glycogen storage disease type IX in children who present with unexplained hepatomegaly and elevated transaminase levels. Furthermore, due to the distinct management of glycogen storage disease type IX compared with glycogen storage disease type I and glycogen storage disease type III, genetic analysis is essential for an accurate diagnosis.

## Introduction

Abnormalities in the enzymes and proteins responsible for glycogen metabolism can lead to a group of metabolic disorders known as glycogen storage diseases (GSDs). These disorders are characterized by an accumulation of glycogen in certain organs, resulting in a range of diseases depending on the type of enzyme and organ affected [1]. The expression of glycogen storage disease type IX (GSD-IX) is linked to the type of phosphorylase kinase (PHK) enzyme deficiency, which is divided into four subtypes: a, b, c, and d. Subtype b is characterized by the involvement of the liver and muscle, while subtypes a, c, and d affect the liver and muscle, respectively [2]. GSD-IX is one of the most common forms of GSD, accounting for a quarter of all cases [3, 4]. It is estimated to occur in 1 in every 100,000 live births worldwide [3].

Ketotic hypoglycemia is a frequent occurrence in all types of GSD, while ketotic normoglycemia is a common finding in GSD-VI and GSD-IX. Genetic testing is a useful tool to differentiate between the two, as they share similar clinical features but have distinct genotypic differences [5]. It is believed that the majority of GSD cases are asymptomatic and do not require treatment. However, some symptomatic cases have been reported to present with short stature, ketogenic hypoglycemia, hepatomegaly, elevated triglyceride levels, and elevated liver transaminases [6]. Maintaining normoglycemia and preventing ketonemia are essential components of the treatment strategy for patients with GSD.

Recent research has indicated that nontherapeutic interventions can have a significant impact on prognosis, with potential implications for developmental delay, daily living functioning, and psychological wellbeing [7]. Dietary therapy has been shown to be effective in reducing the risk of long-term complications, such as chronic ketosis, morning nausea, and growth delay. This has been linked to improved school attendance, increased energy levels, reduced psychological distress, and improved academic performance [7, 8]. Consequently, these patients should abstain from simple sugars, incorporate more complex sugars into their diet, and adhere to a strict high-protein regimen [4]. Here, we report a 4-and-a-half-year-old girl with a novel mutation of the phosphorylase kinase regulatory subunit beta (*PHKB*) gene and describe the diagnostic and therapeutic measures taken during her follow-up. The patient’s legal guardian provided written consent for the publication of this case report. This report was created in accordance with the CARE checklist.

## Case presentation

A 4-year-6-month-old Caucasian girl presented to our pediatric clinic with a diagnosis of glycogen storage disease (GSD). Prior to being referred to our clinic, she had been under the care of pediatric gastroenterologists. Her initial symptoms included chronic abdominal pain and constipation. Upon abdominal examination, her liver was found to be slightly enlarged and coarse to the touch. Initial tests revealed an increase in liver transaminases. During the follow-up, she reported liver symptoms such as itching and jaundice, without any further complications. Consequently, vitamin E and ursodeoxycholic were prescribed as a possible treatment for cholestasis. Given the persistent elevation of liver transaminases, an evaluation of hepatic autoantibodies has been conducted, including AMA-M2, M2-3E, SP-100, LKM-1, LC-1, RO-S2, and PML. No evidence of autoimmune liver disease was found, and the results of the tests were not in favor of Wilson’s diagnosis. The gastroenterologists were unable to make a definitive diagnosis during the follow-up, so they decided to perform a liver biopsy due to the elevated liver transaminases and normal autoantibodies. Spectrophotometry was used to evaluate glycogenesis in the biopsy, which revealed that the liver biopsy showed preserved parenchymal structure with mostly enlarged hepatocytes. These cells had round, uniform nuclei with occasional inclusions, expanded cytoplasm that was clear to wispy pink in color, and sharp borders. Periodic acid–Schiff (PAS) staining confirmed that the cytoplasmic inclusions were glycogen. There were no signs of inflammation or fibrosis.

Following a biopsy, the patient was referred to our endocrinology and metabolic clinic by gastroenterologists with a diagnosis of GSD. This patient was the third child in a family of three, with consanguineous parents. The patient was born via normal vaginal delivery (NVD) at 36 weeks of gestation, weighing 2600 g (between the 5th and 10th percentiles). The patient was breastfed and tolerated it well. At the time of referral, the patient’s weight, height, and head circumference were 14 kg (25th percentile), 100 cm (10th–25th percentile), and 49 cm (15th percentile), respectively. The patient showed no signs of neurodevelopmental delay (NDD) and had achieved all the expected developmental milestones. Motor development was in line with her age. She attended school regularly and achieved satisfactory results. Her medical and family history were both unremarkable, and she was functioning well in a psychosocial sense. On physical examination, the liver was palpable (not coarse), and she had a grade 1a goiter. The laboratory tests demonstrated a hemoglobin (Hb) of 11.6 mg/dl, mean corpuscular volume (MCV)of 88 fl, aspartate transaminase (AST) of 96 U/l, alanine transaminase (ALT) of 88 U/l, alkaline phosphatase (ALP) of 745 U/l, creatinine (Cr) of 0.5 mg/dl, blood urea nitrogen (BUN) of 19 mg/dl, calcium (Ca) of 10.3 mg/dl, phosphorus of 5.6 mg/dl, ferritin of 24 μg/l, and thyroid stimulating hormone of 1.3 m IU/l. Given the diagnosis of GSD, the patient’s condition was managed through dietary measures. As the most common forms of GSD are type I and type III, frequent feedings, a diet rich in complex carbohydrates, such as corn starch supplement, and a lactose restriction were prescribed for the patient. One month later, the doctor recommended laboratory tests such as AST, ALT, creatine phosphokinase (CPK), copper (Cu), prothrombin time (PT), International Normalized Ratio (INR), and ammonia.

After a period of 3 months, the patient was assessed for any signs or symptoms, physical examinations, growth, laboratory tests, and dietary habits. She did not report any particular issue. However, her physical examination revealed hepatomegaly. The laboratory tests showed an AST of 59 U/l, ALT of 86 U/l, CPK of 77 U/l, Cu of 59 μg/dl, PT of 12 seconds, INR of 1.1, and ammonia of 119.4 μg/dl. Additionally, she was not adhering to the prescribed diet. Following the initial appointment, the patient was monitored every 3 months. Unfortunately, over the course of the year-long follow-up, the patient’s condition had not improved; a slight enlargement of the liver was palpable on physical examination, and the patient had not adhered to the prescribed diet. The laboratory results showed that the patient’s lipid profile was within the normal range; however, her liver transaminase levels were elevated. Additionally, her vitamin D level was close to the normal range and her thyroid stimulating hormone (TSH) level was normal. To rule out any secondary causes, such as hypothyroidism, the patient was evaluated. After 1 year, her linear growth had not improved, and her weight and height were 15 kg (10–25th percentile) and 101.5 cm (10–25th percentile), respectively. The physical examination revealed two significant findings: a palpable liver and goiter. Laboratory tests indicated a triglyceride (TG) of 127 mg/dl, high-density lipoprotein (HDL) of 39 mg/dl, low-density lipoprotein (LDL) of 87 mg/dl, total cholesterol (TC) of 144 mg/dl, TSH of 3.35 m IU/L, free T4 of 1.5 μg/dl, vitamin D of 29 ng/ml, Ca of 10 mg/dl, uric acid of 4.5 mg/dl, Cr of 0.6 mg/dl, lactate of 10.2 mg/dl, ferritin of 54 μg/l, AST of 94 U/l, and ALT of 86 U/l. In addition to the existing regimen, vitamin D and vitamin B complex were added. A liver ultrasound and laboratory tests were requested for the next appointment.

At the follow-up visit 1 month later, the patient’s mother reported that the patient had not been using corn starch. On physical examination, the liver was palpable and no other abnormalities were found. Laboratory tests revealed an AST of 60 U/l, ALT of 59 U/l, vitamin D of 70 ng/ml, and lactate of 4.3 mg/dl. The ultrasound report revealed glycogen accumulation in hepatocytes was classified as a grade 2 fatty liver. Fortunately, no adenoma was detected. The liver size was 120 mm, with a coarse and slightly increased echogenicity. The spleen size was normal, measuring 75 mm. The patient was monitored for a year with 3-month follow-up visits, during which her lipid profile, AST, ALT, ALP, vitamin D, GH, IGF-1, and liver ultrasound were assessed. The laboratory results showed a TG of 135 mg/dl, HDL of 47 mg/dl, LDL of 129 mg/dl, TC of 175 mg/dl, AST of 63 U/l, ALT of 88 U/l, ALP of 682 U/l, vitamin D of 17 ng/ml, GH of 7.6 ng/ml, and IGF-1 of 118 ng/ml. The ultrasound report indicated that the size of the liver had grown to 130 mm, and subsequent follow-ups showed that it was continuing to increase. As the patient had reported a loss of appetite and had not been following the prescribed diet, nutritional counseling was recommended to provide a high-carbohydrate diet. In light of the increasing size of the liver, a gastroenterology consultation was requested.

Over the course of the third follow-up, the patient failed to adhere to her prescribed diet, resulting in an unfavorable growth status. Her weight and height were 19 kg (3rd–10th percentile) and 116 cm (10th percentile), respectively. On physical examination, her liver was palpable. Her laboratory tests revealed the following values: TG of 197 mg/dl, HDL of 37 mg/dl, LDL of 99 mg/dl, TC of 162 mg/dl, AST of 62 U/l, ALT of 79 U/l, ferritin of 31.3 μg/l, lactate of 9.6 mg/dl, BUN of 29 mg/dl, Cr of 0.6 mg/dl, CPK of 88 U/l. The liver measured 143 mm in size, and a notable increase in the echo of the liver parenchyma was observed in the liver ultrasound report. Table 1 outlines the diagnostic measures and therapeutic interventions taken.

**Table 1:** Summary of diagnostic measures and therapeutic interventions

Timelines	Diagnostic measures		Therapeutic interventions
	Laboratory test	Other methods	
On referral	AST, ALT, CPK, Cu, PT, INR, ammonia	–	Dietary therapy including corn starch with lactose restriction
During the first-year follow-up	Lipid profile, AST, ALT, TSH, T4, vitamin D, Ca, uric acid, Cr, lactate, ferritin	Liver ultrasound	Dietary therapy including corn starch with lactose restriction, vitamin B complex, vitamin D
During the second-year follow-up	Lipid profile, AST, ALT, ALP, vitamin D, GH, IGF-1	Liver ultrasound	Dietary therapy including corn starch with lactose restriction, vitamin B complex
During the third-year follow-up	Lipid profile, AST, ALT, lactate, ferritin, BUN, Cr, CPK	Liver ultrasound, nutritional consultation, gastroenterologist consultation	Dietary therapy including corn starch with lactose restriction, vitamin B complex, vitamin D
One month later	–	Genetic analysis	Dietary therapy including corn starch with lactose restriction, vitamin B complex, vitamin D, calcium supplement

*TSH* thyroid stimulating hormone, *AST* aspartate transaminase, *ALT* alanine transaminase, *Cu* copper, *CPK* creatine phosphokinase, *PT* prothrombin time, *INR* International Normalized Ratio, *Cr* creatinine, *BUN* blood urea nitrogen, *ALP* alkaline phosphatase

Following a month-long nutritional consultation, a calcium supplementation was added to the patient’s regimen. As the gastroenterologist’s measures proved ineffective, a genetic analysis was requested. Utilizing the whole-exome sequencing (WES) method, the patient’s DNA was examined for a variety of genes, leading to the discovery of a c.C412T (P.Q138x) mutation associated with a defect in the PHK enzyme. According to the Human Gene Mutation Database (HGMD^®^), no reports of this mutation have been made. Furthermore, the American College of Medical Genetics and Genomics (ACMG) has classified it as a variant of uncertain significance (VUS), indicating that it is not a common occurrence. This mutation is believed to be inherited in an autosomal recessive manner, with a homozygous variant located in exon 5 of chromosome 16. Genetic testing was conducted to accurately diagnose the type of GSD the patient was suffering from, as the initial treatment was based on the assumption of GSD-I and GSD-III. However, the treatment for GSD-IX is distinct from that of GSD-I and GSD-III. Accurately determining the type of GSD was of utmost importance. Upon confirming the diagnosis of GSD-IX, it was suggested to consume a lactose-restricted diet with complex carbohydrates, abstain from simple carbohydrates, and adhere to a high protein (more than 2 g/kg/day) and low fat diet. Two and a half years after GSD-IX diagnosis, the patient adhered to the prescribed diet. Her weight and height were 31 kg (25–50th percentile) and 136 cm (25–50th percentile), respectively. Laboratory tests revealed the following values: TG of 128 mg/dl, HDL of 33 mg/dl, LDL of 103 mg/dl, TC of 171 mg/dl, AST of 59 U/l, ALT of 69 U/l, vitamin D of 29 ng/ml, BUN of 29 mg/dl, and Cr of 0.6 mg/dl. She attended school regularly and her performance was acceptable.

## Discussion and conclusion

Patients with PHK enzyme deficiency may present with a range of clinical findings, including growth retardation, hypoglycemia, hepatomegaly, fasting ketosis, muscle cramps, progressive muscle weakness, and myalgia [9]. However, those with *PHKB* mutations tend to have milder symptoms, such as hepatomegaly and mild hypoglycemia, without the muscular symptoms that are typically seen in PHKA2 and PHKG2 mutations [10, 11]. At the outset, our patient exhibited abdominal pain, constipation, and an enlarged liver. Subsequently, Rodríguez-Jiménez *et al*. identified a patient with GSD-IX with a novel PHKA2 variant who presented with a variety of symptoms, such as fasting ketosis, hepatomegaly, muscle weakness, and growth retardation. They highlighted the importance of genetic testing for GSD-IX diagnosis due to the similarity of its symptoms to those of other GSD types [3]. In a rare instance reported by Ramakrishna *et al*., a patient with GSD-IXa and congenital hypothyroidism presented with liver enlargement as the primary symptom. They discussed the WES was found to be a useful tool in diagnosing GSD in patients with clinical suspicion [12]. Jiangwei Zhang *et al*. conducted a study on 17 patients with GSD-IXa and found that 16 and 15 of them had elevated liver transaminases and liver enlargement, respectively [13]. In contrast to our findings, Roscher A *et al*. reported a GSD-IXa case with normal liver transaminase and normal growth condition without treatment [14]. Additionally, Choi *et al*. evaluated PHKA2 mutation in six Korean patients with GSD-IX and found that hepatomegaly was the initial presentation of all patients, with 33% of the patients having short stature—findings that were in agreement with our study [15].

Patients with GSD-IX are characterized by abnormal glycogenolysis and a normal gluconeogenic pathway. This can lead to hepatomegaly and glycogen accumulation in the liver, resulting in hypoglycemia. Fortunately, due to the normal course of gluconeogenesis, severe hypoglycemia is not a symptom of this disease [16, 17]. The body’s energy levels can be affected by fatty acid oxidation, leading to increased ketosis. This can cause difficulty sleeping and irritability in those affected. GSD-IX cases have been observed to experience developmental delays between 6 and 18 months of age, along with hepatomegaly, ketosis, hypoglycemia, elevated lipid levels, and increased liver transaminases [18]. Gastroenterologists often prefer to perform a liver biopsy when they detect hepatomegaly. However, if a diagnosis of GSD-IX is a possibility, a biopsy is not recommended as it could damage the liver’s glycogen stores. In such cases, other methods of diagnosis should be used if possible. The liver biopsies of these patients revealed a mosaic pattern of distended hepatocytes with an accumulation of glycogen, as well as frayed or burst glycogen. Additionally, fibrosis was observed in the periportal region of the lobules, with thin septa [19, 20].

In cases of hypoglycemia and hepatomegaly, a thorough evaluation should be conducted, including liver tests such as PT, albumin, gamma-glutamyltransferase (GGT), AST, ALT enzymes, liver ultrasound, blood glucose (plasma ketone, especially in patients with hypoglycemia), lactate, basic chemistry, urine urganic acid, uric acid, urinalysis, plasma carnitine, complete blood count, creatine kinase, acylcarnitine, and lipid profile. In cases of hypoglycemia where hepatomegaly is not the primary symptom, endocrine tests such as cortisol, insulin, growth hormone, and free fatty acids should be included in the initial diagnostic work-up [21]. Our patient presented with a normal lipid profile, CPK level, serum uric acid and ammonia levels; however, the laboratory profile revealed increased liver transaminases. Rodríguez-Jiménez *et al*. reported that a patient with GSD-IX with a novel PHKA2 variant had increased liver enzymes levels and high total cholesterol [3]. Ramakrishna *et al*. reported a rare case of GSD-IX associated with congenital hypothyroidism. The patient exhibited elevated levels of both serum glutamic-oxaloacetic transaminase (SGOT) and serum glutamic-pyruvic transaminase (SGPT), high GGT, high triglycerides, hepatomegaly, and altered echogenicity of liver ultrasound, which were similar to the findings of our patient [12]. In a study of six Korean patients with GSD-IX, elevated liver transaminases were the initial findings in all patients, which is consistent with our research [15]. Transaminase levels are a common occurrence in patients with GSD-IX [14, 22]. Patients with normal-to-elevated GGT levels and elevated lactate levels may benefit from a metabolic profile assessment. High TC and TG levels [14], normal or partially elevated creatine kinase (CK) levels due to protein deficiency, and normal lactate and uric acid levels may be observed. Additionally, an increased liver size and increased echogenicity in liver ultrasound findings have been reported in these patients [22]. Laboratory tests such as PT, albumin, INR, transaminase, GGT, and ALP should be used to both initially diagnose and monitor the patient’s condition over the course of 3–12 months. Echocardiographic evaluations should also be conducted every 5 years to assess cardiac involvement in GSD-IX cases with muscle enzyme deficiency [23]. As the prognosis of this disease is not entirely clear, it is important to investigate this profile even if the patient is in metabolic control and has an adequate clinical status [11].

Recent studies with long-term follow-up of GSD-IX patients have revealed that clinical findings such as hepatomegaly and increased transaminases can be reversed over time, and the developmental status of patients may even normalize in adulthood. Unfortunately, some GSD-IX cases do not have a positive prognosis and can even progress to liver cirrhosis [7, 24, 25]. The role of regimens in patients with GSD-IX is to help prevent the development of serious symptoms such as hepatomegaly, hypoglycemia, ketosis, cirrhosis, short stature, and growth retardation. To this end, it is recommended that these patients eat small, frequent meals throughout the day and avoid fasting whenever possible [11]. Treatment of these patients should focus on frequent, nonbulky meals, with minimal use of simple sugars and a high-protein diet (2–3 g/kg). The consumption of uncooked cornstarch is suggested to help avoid episodes of hypoglycemia that come with ketosis in these patients. Uncooked cornstarch and protein have been found to be effective in preventing these occurrences during the night [26]. Although this condition may seem harmless, it can lead to long-term complications [8]. Studies have demonstrated that providing adequate nutrition to these patients can lead to improved growth and laboratory results [26]. It is strongly advised to take aggressive action when transaminase levels and postprandial lactate levels are elevated, as this may be a sign of cirrhosis progression [8]. Given the mild and varied clinical manifestations of the patients, it is possible to overlook the diagnosis. Consequently, it is essential to keep a close watch on the rise of liver transaminases and the size of the liver until a definitive diagnosis is made. Genetic testing can be a useful tool in diagnosing these patients.

## Data Availability

The datasets used and/or analyzed during the current study are available from the corresponding author on reasonable request.

## Abbreviations

GSD: Glycogen storage disease
PHKB: Phosphorylase kinase regulatory subunit beta
PAS: Periodic acid–Schiff
NVD: Normal vaginal delivery
NDD: Neurodevelopmental delay
Hb: Hemoglobin
MCV: Mean corpuscular volume
AST: Aspartate transaminase
ALT: Alanine transaminase
ALP: Alkaline phosphatase
Cr: Creatinine
BUN: Blood urea nitrogen
Ca: Calcium
TSH: Thyroid stimulating hormone
TG: Triglyceride
HDL: High-density lipoprotein
LDL: Low-density lipoprotein
TC: Total cholesterol
CPK: Creatine phosphokinase
Cu: Copper
PT: Prothrombin time
INR: International normalized ratio
WES: Whole exome sequencing
HGMD: Human gene mutation database
VUS: Variant of uncertain
ACMG: American College of Medical Genetics and Genomics
PHK: Phosphorylase kinase
GGT: Gamma-glutamyltransferase
CK: Creatine kinase
SGOT: Serum glutamic-oxaloacetic transaminase
SGPT: Serum glutamic-pyruvic transaminase
